# Fucosterol Protects against Concanavalin A-Induced Acute Liver Injury: Focus on P38 MAPK/NF-*κ*B Pathway Activity

**DOI:** 10.1155/2018/2824139

**Published:** 2018-07-17

**Authors:** Wenhui Mo, Chengfen Wang, Jingjing Li, Kan Chen, Yujing Xia, Sainan Li, Ling Xu, Xiya Lu, Wenwen Wang, Chuanyong Guo

**Affiliations:** ^1^Department of Gastroenterology, Shanghai Tenth People's Hospital, Tongji University School of Medicine, Shanghai 200072, China; ^2^Putuo District People's Hospital, Tongji University School of Medicine, Shanghai 200060, China; ^3^Department of Gastroenterology, Shanghai Tongren Hospital, Shanghai Jiaotong University School of Medicine, Shanghai 200336, China

## Abstract

**Objective:**

Fucosterol is derived from the brown alga *Eisenia bicyclis* and has various biological activities, including antioxidant, anticancer, and antidiabetic properties. The aim of this study was to investigate the protective effects of fucosterol pretreatment on Concanavalin A- (ConA-) induced acute liver injury in mice, and to understand its molecular mechanisms.

**Materials and Methods:**

Acute liver injury was induced in BALB/c mice by ConA (25 mg/kg), and fucosterol (dissolved in 2% DMSO) was orally administered daily at doses of 25, 50, and 100 mg/kg. The levels of hepatic necrosis, apoptosis, and autophagy associated with inflammatory cytokines were measured at 2, 8, and 24 h.

**Results:**

Fucosterol attenuated serum liver enzyme levels and hepatic necrosis and apoptosis induced by TNF-*α*, IL-6, and IL-1*β*. Fucosterol also inhibited apoptosis and autophagy by upregulating Bcl-2, which decreased levels of functional Bax and Beclin-1. Furthermore, reduced P38 MAPK and NF-*κ*B signaling were accompanied by PPAR*γ* activation.

**Conclusion:**

This study showed that fucosterol could alleviate acute liver injury induced by ConA by inhibiting P38 MAPK/PPAR*γ*/NF-*κ*B signaling. These findings highlight that fucosterol is a promising potential therapeutic agent for acute liver injury.

## 1. Introduction

The liver is the largest organ within the abdominal cavity and undertakes important physiological processes. Liver injury is the basis of acute liver failure and is primarily caused by viral infections, drugs, food additives, alcohol, and radioactive damage [1]. Currently, the etiology of acute liver injury is unclear and treatment with glucocorticoids or azathioprine often results in severe side effects [2]. Therefore, it is important to establish an animal model to screen effective drugs.

Immune responses are mediated by different cells and cytokines in different injury models. Concanavalin A (ConA) is a plant agglutinin extracted from Brazilian rubber beans that was firstly used to study liver injury by Tiegs et al. [3]. ConA can modify the major histocompatibility complex (MHC) structure to produce inflammatory reactions, by activating macrophages and CD4^+^ T cells, which release TNF-*α*, IL-1*β*, IL-6, and other inflammatory factors that damage hepatic cells [4]. Thus, ConA treatment is a good simulation of the clinical onset of AIH and viral hepatitis. The model also has the advantages of utilizing a simple extraction and causing liver-specific damage (injury to other organs is not obvious), thus providing a reliable animal model for clinical research in basic immunology [5]. The ConA-induced inflammatory process is mediated by a series of endogenous inflammatory factors. Peroxisome proliferator-activated receptors (PPARs) are ligand-activated transcription factors that regulate lipid metabolism, blood pressure, cell growth, and differentiation [6–8]. In recent years, PPARs have been found to play important roles in the pathogenesis of inflammation [9–12]. PPARs are divided into three subtypes, PPAR*α*, PPAR*β*, and PPAR*γ*, which all regulate inflammatory responses [11]. PPAR*γ* is especially well characterized because of its close relation with inflammatory signaling pathways such as NF-*κ*B, activator protein 1 (AP-1), and JAK/STAT [13].

Fucosterol is separated and purified by silica gel column chromatography from the ethanol extract of brown algae [14] and has many pharmacological effects on various human ailments, such as diabetes, cancer, inflammation, and oxidation. Jung et al. showed that fucosterol could inhibit LPS-induced nitric oxide and *tert*-butylhydroperoxide-induced reactive oxygen species along with suppressing inducible nitric oxide synthase and cyclooxygenase-2 [15]. Yoo et al. showed that fucosterol inhibited the expression of inducible nitric oxide synthase, TNF-*α*, and IL-6, as well as suppressed the NF-*κ*B and P38 MAPK pathways in LPS-induced RAW264.7 macrophages [16]. However, the *in vivo* effects of fucosterol are still unclear for acute liver injury. We hypothesized that fucosterol may have a protective effect on ConA-induced inflammation, and that its anti-inflammatory mechanism could be associated with NF-*κ*B and PPAR*γ* in mice.

## 2. Materials and Methods

### 2.1. Animals

Male BALB/c mice weighing 20–25 g (6–8 weeks old) were purchased from Shanghai SLAC Laboratory Animal Co. Ltd. (Shanghai, China) and housed in a clean room at 23 ± 2°C, 50% humidity, with a 12 h light/dark cycle. Animals were permitted free access to food and water, and all experiments were performed according to the National Institutes of Health Guidelines for the Care and Use of Laboratory Animals. The experiments were also approved by the Animal Care and Use Committee of Tongji University, Shanghai, China.

### 2.2. Reagents

Fucosterol and anisomycin were purchased from Sigma-Aldrich (St. Louis, MO, USA) and dissolved in 2% DMSO. Antibodies purchased from Cell Signaling Technology (Danvers, MA, USA) included TNF-*α*, PPAR*γ*, RXR*α*, IL-6, IL-1*β*, LC3, NF-*κ*B p65, P38 MAPK, p-P38 MAPK, JNK, p-JNK, ERK, and p-ERK. Antibodies purchased from Proteintech (Chicago, IL, USA) included Beclin-1, Bax, Bcl-2, P62, and *β*-actin. Liver enzymes were detected and analyzed using microplate test kits (Nanjing Jiancheng Biotech, Nanjing, China) and the RNA PCR kit was purchased from Takara Biotechnology (Dalian, China). Enzyme-linked immunosorbent assay (ELISA) kits for TNF-*α*, IL-6, and IL-1*β* were acquired from eBioscience (San Diego, CA, USA). A terminal deoxynucleotidyl transferase dUTP nick-end labeling (TUNEL) apoptosis assay kit was purchased from Roche (Basel, Switzerland).

### 2.3. Experimental Design

Mice were housed in a warm humid environment and treated with or without fucosterol by gavage for 3 d. Then, ConA was dissolved in normal saline solution and injected via tail vein to induce acute liver injury as previously demonstrated [17]. All mice were randomly distributed to different groups as follows: group I, normal (saline) group (*n* = 6); group II, control (2% DMSO) group (*n* = 18); group III, fucosterol (100 mg/kg) group (*n* = 18); group IV, ConA (25 mg/kg) group (*n* = 18); group V, treatment group (*n* = 54): ConA (25 mg/kg) + fucosterol (25, 50, or 100 mg/kg).

All mice in groups I, II, and III were sacrificed after 3 d, while six mice were randomly selected from groups IV to V (6 from each dose) and sacrificed 2, 8, and 24 h after ConA injection. Blood and liver tissues were collected for further analysis. The results of preliminary experiments showed no significant differences in biochemical or pathological indicators of liver disease between groups I and II, so group II was selected as the experimental control group.

### 2.4. Serum Enzymes and Cytokines

Serum was separated by centrifugation at 4500 rpm at 4°C for 10 min. The supernatant was used to detect alanine aminotransferase (ALT), aspartate aminotransferase (AST), and cytokines including TNF-*α*, IL-6, and IL-1*β* according to the manufacturer's protocols.

### 2.5. Pathological Assessments

Fresh liver tissue was washed with saline, embedded in 4% paraffin, and then cut into 5 *μ*m sections. After drying for 2 h in an incubator, nuclei and cytoplasm were stained with hematoxylin and eosin (HE). Specific pathological changes were observed under a light microscope.

### 2.6. Western Blot Analysis

Frozen liver tissue (100 mg) was homogenized in 600 *μ*L of radioimmunoprecipitation assay (RIPA) lysis buffer containing protease inhibitors and phenylmethane-sulfonyl fluoride (PMSF) by incubating on ice for 40 min. Supernatant was collected for bicinchoninic acid (BCA) assay after centrifugation at 12,000 rpm for 15 min. Equal amounts of protein were separated by SDS-PAGE using standard techniques, and separated proteins were transferred to activated polyvinylidene fluoride membranes. Membranes were blocked using 5% nonfat dried milk, and primary antibodies (CST, 1 : 1000; Proteintech, 1 : 500) diluted in blocking buffer were incubated at 4°C overnight. The next day, membranes were washed with phosphate-buffered saline containing 0.1% Tween 20 and incubated with anti-rabbit or anti-mouse IgG secondary antibodies (1 : 2000) for 1 h at 37°C. The Odyssey two-color infrared laser imaging system (LI-COR Biosciences, Lincoln, NE, USA) was used to analyze band densities with *β*-actin as the internal loading control.

### 2.7. Quantitative Real-Time PCR (qRT-PCR)

Total RNA was extracted from approximately 50 mg of liver tissue using TRIzol, chloroform, and isopropyl alcohol. After determining RNA concentration, a reverse transcription kit was used to transcribe RNA into cDNA. SYBR Green qRT-PCR was performed to determine gene expression levels using a 7900HT fast real-time PCR system (Applied Biosystems, Foster City, CA, USA) according to the provided protocol. Levels of target gene and *β*-actin were compared on the basis of the solubility curve. Primers used in qRT-PCR experiments are shown in Table 1.

### 2.8. Immunohistochemistry

After the paraffin sections were dewaxed in dimethylbenzene and rehydrated through a graded series of alcohol, antigen retrieval was performed with citric acid-hydrogen phosphate two sodium buffers. Then, 3% hydrogen peroxide solution was added for 5 min to block endogenous peroxidase activity. After blocking with 5% bovine serum albumin at 37°C for 20 min and at room temperature for 10 min, antibodies (CST, 1 : 100; Proteintech, 1 : 50) were incubated with the sections overnight in a wet box at 4°C. The next day, the slices were washed with PBS and incubated with goat anti-rabbit secondary antibody for 30 min at room temperature. A diaminobenzidine kit was used to colorize the staining for imaging under a light microscope.

### 2.9. Transmission Electron Microscopy

Liver tissue was perfused with 2% glutaraldehyde buffered with 0.2 mmol/L cacodylate for 4 h. Sections were observed by transmission electron microscopy (JEM-1230; JEOL, Tokyo, Japan) to show autophagosomes after postfixation in 1% osmium tetroxide for 1 h.

### 2.10. Cell Culture and CCK8 Assay

The normal LO2 cells were cultured in RPMI-1640 culture medium (Thermo Fisher Scientific (China) Co. Ltd.) supplemented with 10% fetal bovine serum (Hyclone™ Fetal Bovine Serum, South American Origin), 100 U/mL of penicillin, and 100 g/mL of streptomycin (Gibco, Canada) in a humidified incubator at 37°C under 5% CO_2_. The cells were plated at a density 2 × 10^4^ cells/well in 96-well plates (100 *μ*L of medium per well). The concentration of fucosterol was 20 *μ*M and the anisomycin concentration was 0.1 *μ*M. Cell viability was measured with the CCK8 assay at a wavelength of 450 nm. The LO2 were divided into four groups:

1. Control group: no treatment
2. Anisomycin group: treated with anisomycin diluted in DMSO at a concentration of 0.1 *μ*M
3. Fucosterol group: treated with fucosterol diluted in DMSO at a concentration of 20 *μ*M
4. F + A group: treated with fucosterol (20 *μ*M) and anisomycin (0.1 *μ*M)

### 2.11. Statistical Analysis

All statistical analyses were calculated using SPSS V22.0 (IBM, Armonk, NY, USA). Experimental data are presented as the mean ± standard deviation and were compared by one-way analysis of variance using the Student-Newman-Keuls method. Differences were considered significant at *p* < 0.05. Histograms were created using GraphPad Prism v6.0 (GraphPad, San Diego, CA, USA).

## 3. Results

### 3.1. Fucosterol Pretreatment Ameliorated ConA-Induced Acute Liver Necrosis

**Figure 1:**
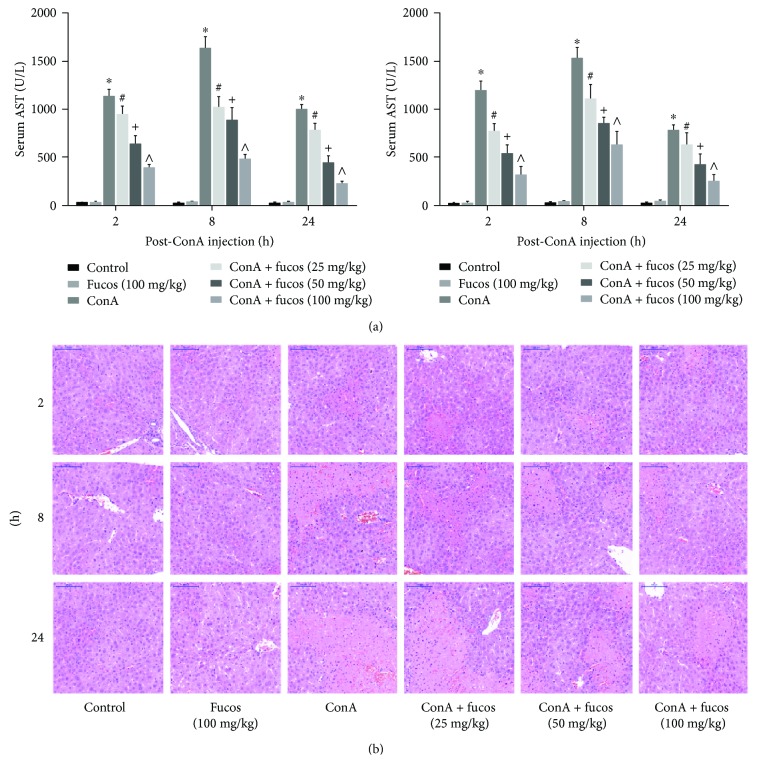
Effects of fucosterol on serum liver enzymes and acute liver injury pathology. (a) Serum ALT and AST levels 2, 8, and 24 h after ConA injection. Data are expressed as mean ± SD (*n* = 6, ^∗^*P* < 0.05 for ConA versus control, ^#^*P* < 0.05 for ConA + fucosterol (25 mg/kg) versus ConA, ^+^*P* < 0.05 for ConA + fucosterol (50 mg/kg) versus ConA + fucosterol (25 mg/kg), and ^∧^*P* < 0.05 for ConA + fucosterol (100 mg/kg) versus ConA + fucosterol (50 mg/kg)). (b) Hematoxylin and eosin staining of liver sections. Necrotic areas were imaged by digital microscopy; original magnification: 200x.

Transaminases are primarily stored in hepatocytes and are the most sensitive indicators of hepatocyte necrosis.  Figure 1(a) shows serum ALT and AST levels of mice from different groups, and these results indicated that fucosterol did not cause changes in liver enzymes at 2, 8, or 24 h. However, ALT and AST levels increased rapidly after ConA injection, peaking at 8 h. Fucosterol pretreatment decreased this trend in a dose-dependent manner at different time points. Then we examined nuclei and cytoplasm through HE staining, which visually indicated the extent of liver cell damage (Figure 1(b)). These results showed that liver cells in the ConA group demonstrated nuclear condensation and fragmentation, and the cell outline disappeared and was replaced by an amorphous, red-colored granular coagulation or liquefied substance. After fucosterol pretreatment, the area of necrosis decreased significantly with increasing drug concentration, and the most severe phenotypes were only present at 24 h. This indicated that the drug significantly reduced ConA-induced acute liver injury at 2, 8, and 24 h. As liver injury peaked at 8 h, we selected this time point for subsequent studies.

### 3.2. Fucosterol Inhibited Apoptosis in the ConA-Induced Acute Liver Injury Model

**Figure 2:**
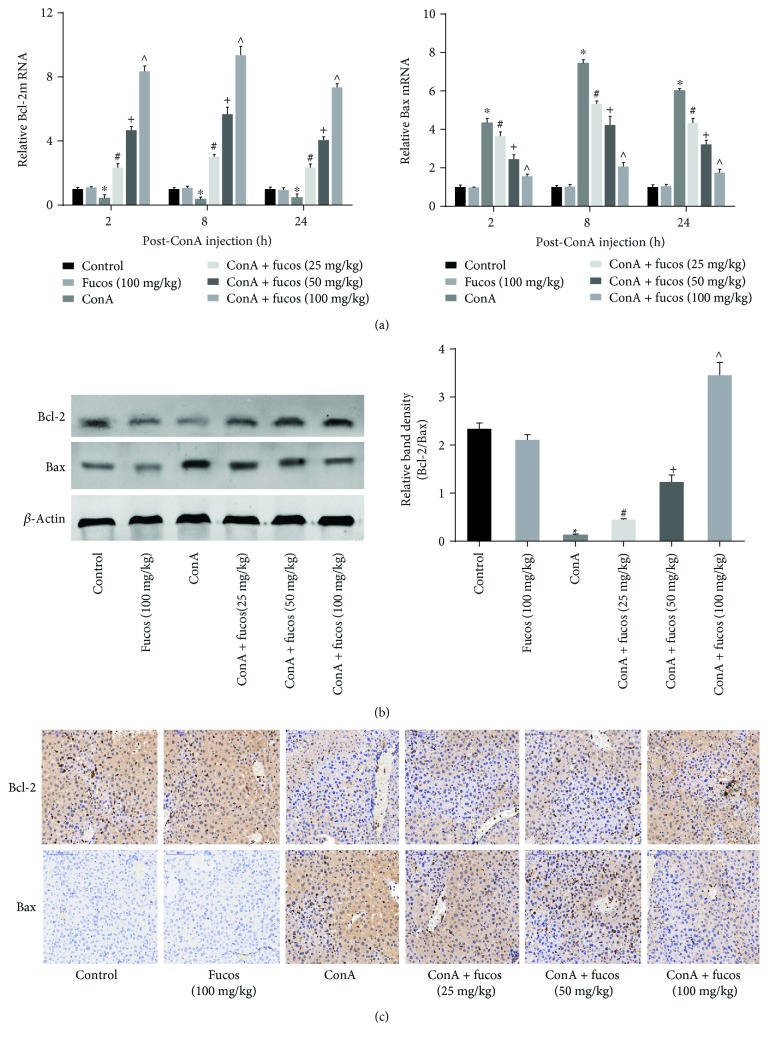
Effects of fucosterol on apoptosis in the acute liver injury model. (a) The expression of Bcl-2 and Bax mRNA were evaluated by quantitative real-time PCR. (b) Western blot and analysis of Bcl-2 and Bax protein levels. (c) Immunohistochemistry was used to detect Bcl-2 and Bax expression at 8 h ConA treatment. Original magnification: 200x. Data are expressed as mean ± SD (*n* = 6, ^∗^*P* < 0.05 for ConA versus control, ^#^*P* < 0.05 for ConA + fucosterol (25 mg/kg) versus ConA, ^+^*P* < 0.05 for ConA + fucosterol (50 mg/kg) versus ConA + fucosterol (25 mg/kg), and ^∧^*P* < 0.05 for ConA + fucosterol (100 mg/kg) versus ConA + fucosterol (50 mg/kg)).

In addition to necrosis, apoptosis and autophagy are also important mechanisms of cell death. Therefore, we chose 8 h ConA treatment to further explore the effects fucosterol on apoptosis. Transcription of the antiapoptotic gene Bcl-2 was significantly decreased in the model group, whereas the proapoptotic Bax was increased. Fucosterol treatment significantly increased Bcl-2 transcription in a dose-dependent manner compared with the ConA group. In contrast, Bax was decreased in the fucosterol treatment groups (Figure 2(a)). Next, we detected Bcl-2 and Bax protein levels and localization in tissues using Western blot and immunohistochemistry, respectively (Figures 2(b) and 2(c)). Expression of Bcl-2 and Bax proteins were consistent with the mRNA results.

### 3.3. Fucosterol Reduced Autophagy Levels in the ConA-Induced Acute Liver Injury Model

**Figure 3:**
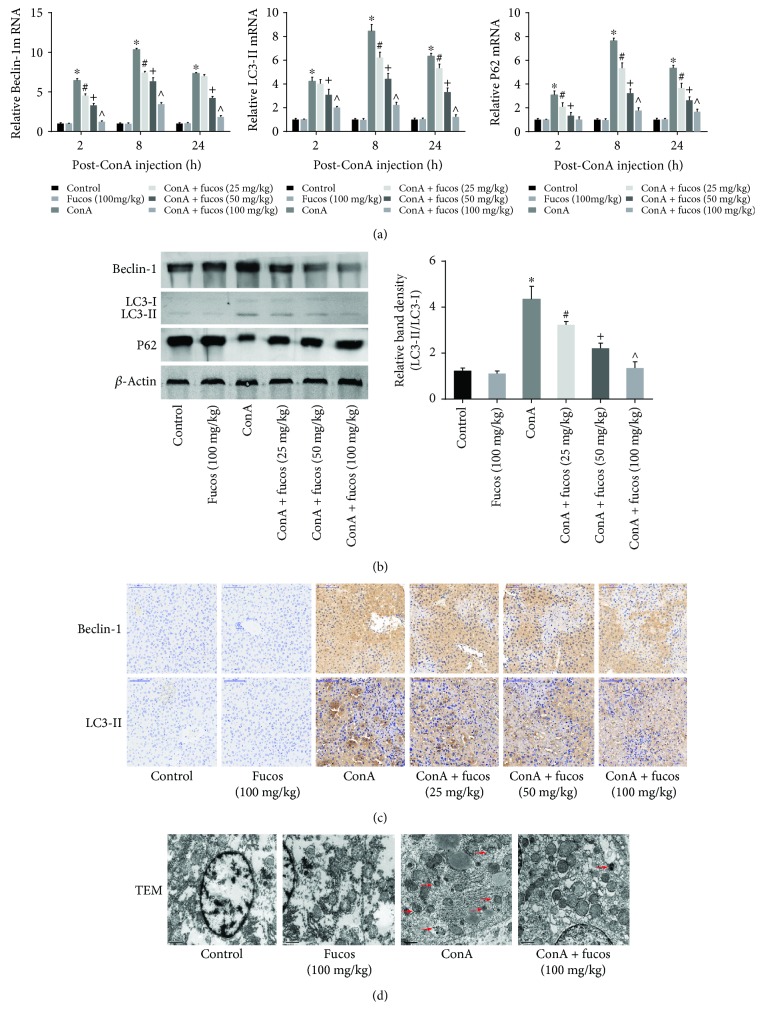
Effects of fucosterol on autophagy during acute liver injury. (a) The expression of Beclin-1, LC3-II, and P62 mRNA were evaluated by real-time PCR. (b) Western blot and analysis of Beclin-1, LC3-II, and P62 proteins. (c) Immunohistochemistry was used to detect the level of Beclin-1, LC3-II, and P62 expression at 8 h. Original magnification: 200x. (d) Autophagosome formation was detected in liver tissues with transmission electron microscopy at 8 h (magnification, ×10,000). Arrows indicate autophagosomes. Data are expressed as mean ± SD (*n* = 6, ^∗^*P* < 0.05 for ConA versus control, ^#^*P* < 0.05 for ConA + fucosterol (25 mg/kg) versus ConA, ^+^*P* < 0.05 for ConA + fucosterol (50 mg/kg) versus ConA + fucosterol (25 mg/kg), and ^∧^*P* < 0.05 for ConA + fucosterol (100 mg/kg) versus ConA + fucosterol (50 mg/kg)).

Autophagy is a recently acknowledged cellular phenomenon that is related to programmed cell death. Thus, we also assessed autophagy levels, using Beclin-1, LC3, and P62 as autophagy markers. At the mRNA and protein level, Beclin-1 and LC-3 II were activated and showed increased expression in the ConA group, while autophagy levels were decreased after fucosterol treatment, which was most apparent at the maximum concentration (Figures 3(a) and 3(b)). Fucosterol had no obvious influence on autophagy in untreated normal animals. Inhibiting autophagy caused an accumulation of P62, so contrary to Beclin-1 and LC-3 II, P62 showed increased expression after fucosterol treatment. Furthermore, we performed immunohistochemical staining to directly observe autophagy levels *in vivo*, and the results were consistent with the expression data (Figure 3(c)). Autophagosomes with a bilayer membrane and autolysosomes after cytoplasmic degradation are important indicators of autophagy. We used transmission electron microscopy to show that the number of autophagosomes and autolysosomes increased after ConA injection, and that fucosterol inhibited this trend (Figure 3(d)). These results suggest that fucosterol inhibited autophagy and reduced hepatocyte death *in vivo*.

### 3.4. Fucosterol Inhibited the Release of Inflammatory Factors and NF-*κ*B p65 but Activated PPAR*γ* in the ConA-Induced Acute Liver Injury Model

**Figure 4:**
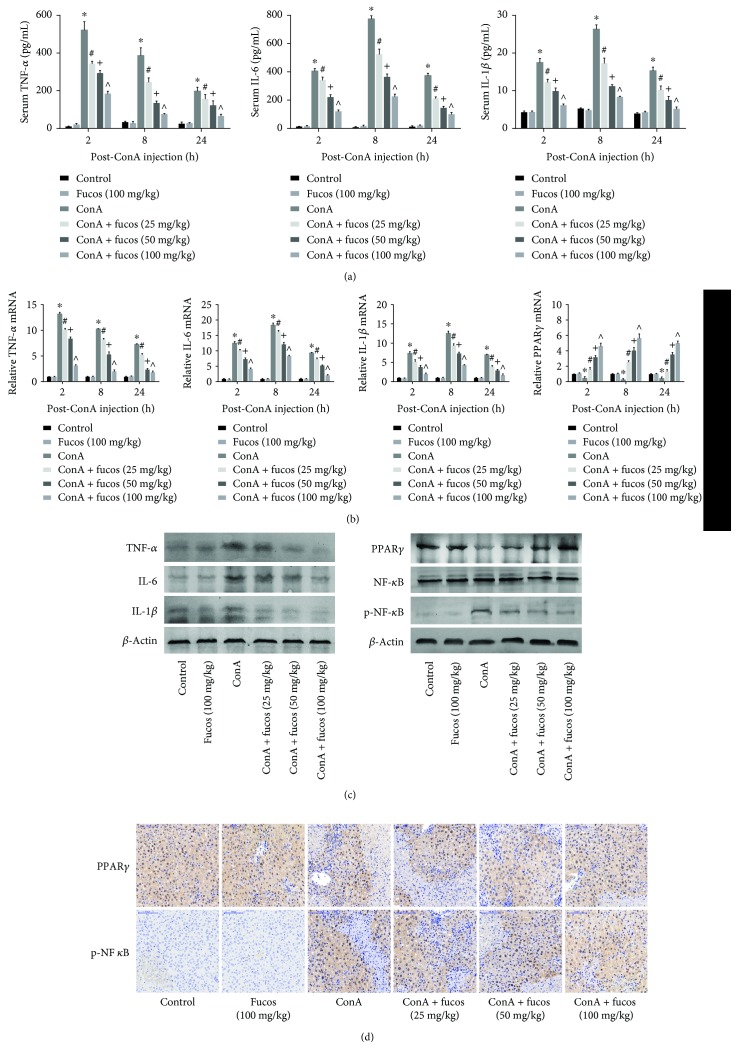
Effects of fucosterol on inflammatory factors in the acute liver injury model. (a) Serum levels of TNF-*α*, IL-6, and IL-1*β* 2, 8, and 24 h after ConA injection. (b) TNF-*α*, IL-6, IL-1*β*, and PPAR*γ* mRNA levels were evaluated by quantitative real-time PCR. (c) Western blot and analysis of TNF-*α*, IL-6, IL-1*β*, PPAR*γ*, NF-*κ*B p65, and p-NF-*κ*B p65 protein expression. (d) Immunohistochemistry was used to detect PPAR*γ* and p-NF-κB p65 expression at 8 h. Original magnification: 200x. Data are expressed as mean ± SD (*n* = 6, ^∗^*P* < 0.05 for ConA versus control, ^#^*P* < 0.05 for ConA + fucosterol (25 mg/kg) versus ConA, ^+^*P* < 0.05 for ConA + fucosterol (50 mg/kg) versus ConA + fucosterol (25 mg/kg), and ^∧^*P* < 0.05 for ConA + fucosterol (100 mg/kg) versus ConA + fucosterol (50 mg/kg)).

Inflammatory factors can activate neutrophils and lymphocytes causing necrosis or activate other signaling pathways that lead to apoptosis and autophagy. Therefore, inflammatory factors are also an important parameter in assessing cell damage. We evaluated TNF-*α*, IL-6, and IL-1*β*, the major mediators in ConA-induced liver injury, and found that their expression was consistent with the extent of necrosis, apoptosis, and autophagy. Serum TNF-*α*, IL-6, and IL-1*β* increased markedly after ConA treatment, while fucosterol pretreatment inhibited their release, and showed the greatest effect at the maximum concentration (Figure 4(a)). mRNA and protein levels of these inflammatory factors were also significantly decreased in liver tissue from the fucosterol treatment group compared with the ConA group, and the changes were statistically significant and in a concentration-dependent manner (Figure 4(b)). We further explored NF-*κ*B p65 and its upstream pathway PPAR*γ*, which play a major role in the release of inflammatory cytokines. These results showed that increased TNF-*α*, IL-6, and IL-1*β* release was associated with increased NF-*κ*B p65 phosphorylation and decreased PPAR*γ* expression. Immunohistochemistry showed a consistent trend with the Western blot data (Figure 4(c)). These data suggested that fucosterol may activate PPAR*γ*, thereby inhibiting the NF-*κ*B p65 pathway, which reduces the expression of inflammatory factors and subsequent necrosis and apoptosis.

### 3.5. Fucosterol Inhibited P38 MAPK Phosphorylation but Not JNK or ERK

**Figure 5:**
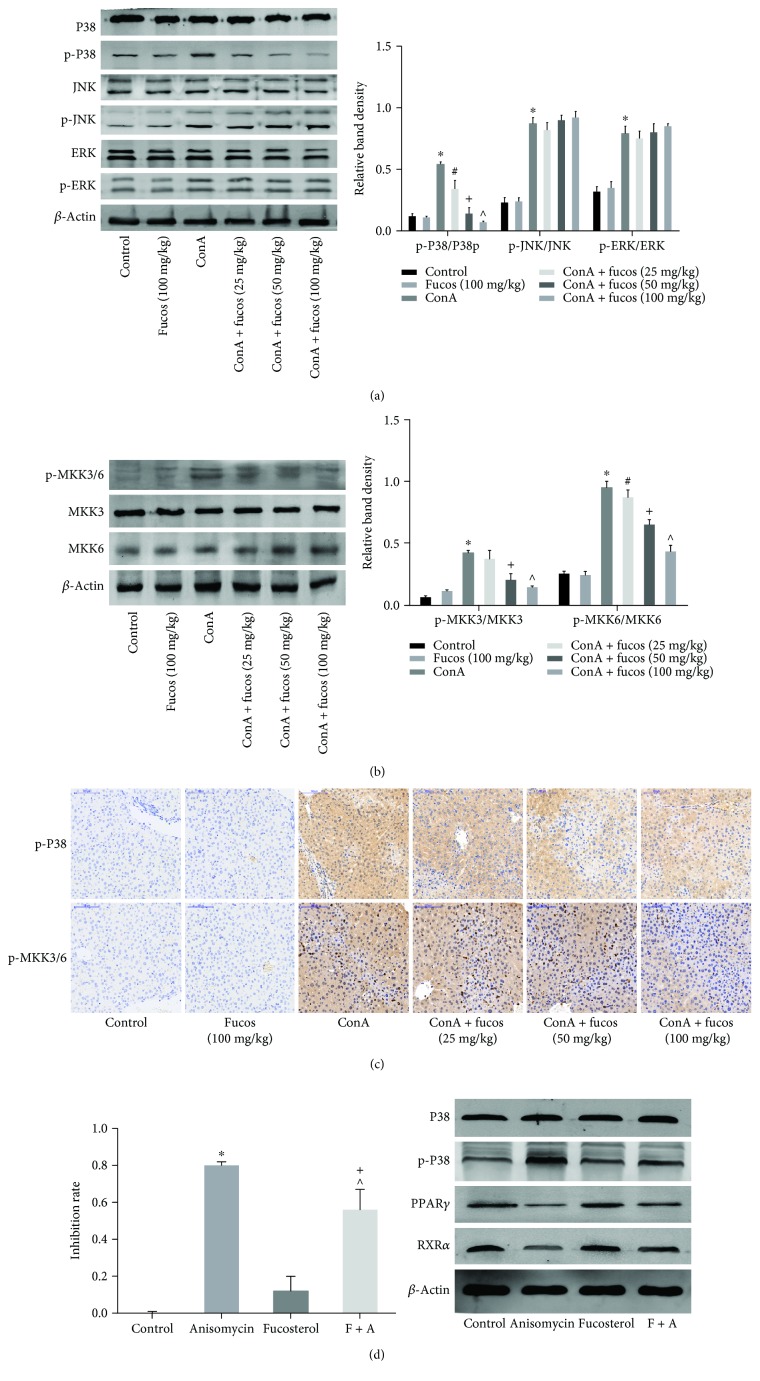
Effect of fucosterol on MAPK family activation. (a) Western blot and analysis of P38, p-P38, JNK, p-JNK, ERK, and p-ERK. (b) Western blot and analysis of p-MKK3/6, MKK3 and MKK6. (c) Immunohistochemistry was used to detect p-P38 and p-MKK3/6 levels at 8 h. Original magnification: 200x. (d) The proliferation of LO2 with CCK8 assay and Western blot and analysis of P38, p-P38, PPAR*γ*, and RXR*α*. Data are expressed as mean ± SD (*n* = 6, ^∗^*P* < 0.05 for ConA versus control, ^#^*P* < 0.05 for ConA + fucosterol (25 mg/kg) versus ConA, ^+^*P* < 0.05 for ConA + fucosterol (50 mg/kg) versus ConA + fucosterol (25 mg/kg), and ^∧^*P* < 0.05 for ConA + fucosterol (100 mg/kg) versus ConA + fucosterol (50 mg/kg)).

We next sought to determine how fucosterol activated PPAR*γ*, testing the hypothesis that the target may be a MAPK family member, based on these being upstream of PPAR*γ* and on the pharmacological properties of fucosterol. Therefore, we examined the levels of P38 MAPK, JNK, and ERK phosphorylation. Western blot results showed that although ConA elevated phosphorylation levels of all MAPK family members, fucosterol only reduced the activated form of P38 MAPK, and had no effect on phosphorylated JNK or ERK (Figure 5(a)). Next, we examined p-P38 MAPK levels in liver tissue using immunohistochemistry, which confirmed the above results (Figure 5(b)). Furthermore, we investigated MKK3/6 activity, which is upstream of P38 MAPK, and these results were consistent with our hypothesis. In the ConA group, levels of phosphorylated MKK3/6 increased significantly, but with increasing fucosterol dosage, the trend of p-MKK3/6 and p-P38 MAPK decreased significantly (Figure 5(c)). The P38 activator anisomycin was used to demonstrate the direct effect of fucosterol on P38 MAPK/PPAR*γ*. After being treated for 48 h, the protein of cells were extracted to evaluate the expression of P38 MAPK, PPAR*γ*, and RXR*α*. However, the use of a single drug did not have a significant effect on all the MAPK pathways. Therefore, fucosterol may act by inhibiting P38 MAPK activation, but had no effect on JNK or ERK.

## 4. Discussion

The incidence of AIP has increased worldwide; thus, it is prudent to explore the potential activity of natural products that have low side effects to develop more effective therapeutic strategies [18–21]. Fucosterol derived from the brown alga *Eisenia bicyclis* is the most abundant phytosterol and has various biological activities, including antioxidant, anticancer, and antidiabetic properties [14]. Recently, fucosterol was found to have an anti-inflammatory effect but its mechanism of liver protection remains unclear [15, 22].

After tail vein injection, ConA can activate T cells and macrophages, exert cytotoxic effects through perforin and granzyme and cause cell death that can be divided into necrosis, apoptosis, and autophagy according to cell morphology. FasL expression is induced during this process, which mediates TNF-*α*, IL-6, and IL-1*β* secretion, causing autophagy-associated necrosis and apoptosis [23–25]. Therefore, if a therapeutic agent could effectively inhibit these three cell death pathways, it could protect liver cells. First, we detected the effect of fucosterol on necrosis. ALT and AST release peaked 8 h after ConA injection, which was explained by the membrane permeability of liver cells that caused ALT and AST release to blood. Fucosterol pretreatment reduced transaminase levels and had a greater effect under higher doses, indicating that the drug can effectively reduce liver cell necrosis. The pathology directly reflects the edema and necrosis in the tissues, and also proves the effect. These results were consistent with the evidence provided by Yoo et al. and Li et al. [16, 26]. Second, we investigated Bcl-2 and Bax expression, which are important indicators of endogenous apoptosis. Bcl-2 and Bax can form homologous or heterologous dimers to regulate apoptosis. Bcl-2, an antiapoptotic protein, showed reduced expression after ConA treatment, while Bax showed increased expression. Fucosterol treatment changed the trend of both these markers [27]. Thus, fucosterol can increase the ratio of Bcl-2/Bax causing decreased Bax/Bax homologous dimers, which normally can lead to cytochrome *c* and AIF release by translocation from the cytosol to mitochondria. At the same time, increased Bcl-2 expression can reduce apoptosis independent of Bax. Pattingre et al. showed that Bcl-2 can downregulate Beclin-1-dependent autophagic cell death [28]. Free Beclin-1 can lead to the conversion of LC3-I to LC3-II, which increases the occurrence of autophagic vesicles and reduces P62 accumulation [29]. Thus, fucosterol can protect liver cells by inhibiting necrosis, apoptosis, and autophagy.

The release of inflammatory factors plays an important role in ConA-induced acute liver injury. Our detection of inflammatory factors showed that the major inducers, such as TNF-*α*, IL-6, and IL-1*β*, were markedly decreased in a dose-dependent manner after fucosterol treatment. While, the results from Sun et al. suggested that fucosterol protected HaCaT cells against CoCl_2_-induced cytotoxicity and inflammatory responses by suppressing HIF1-*α* [22], Li et al. showed that fucosterol attenuates lipopolysaccharide-induced acute lung injury by inhibiting NF-*κ*B activation [26]. Therefore, we tested whether fucosterol activity in our model was mediated acting on the proinflammatory signaling pathways, such as PPAR*γ*/NF-*κ*B, MAPK, and JAK/STAT. We tested these pathways because Feng et al. provided evidence that many natural products regulate PPAR*γ* [30], and Fuenzalida et al. and Mansour et al. also highlighted the beneficial effects of curcumin and 15-deoxy-Δ12,14-PGJ2, which are mediated by upregulating PPAR*γ* activation [31, 32].

**Figure 6:**
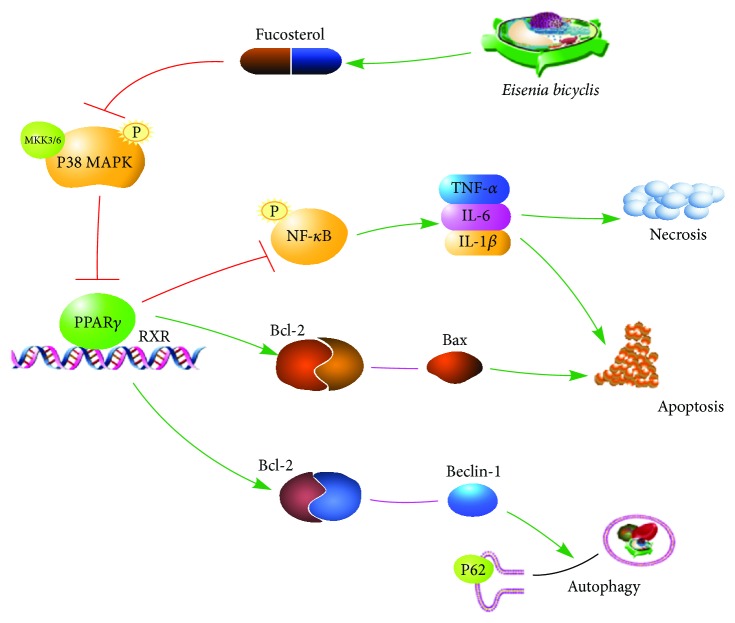
Mechanism of fucosterol action. In ConA-induced acute liver injury, fucosterol decreased P38 MAPK phosphorylation and contributed to the increased PPAR*γ* transcriptional activity. Active PPAR*γ* reduced the release of inflammatory factors that cause necrosis and apoptosis by inhibiting the NF-*κ*B pathway. Additionally, Bcl-2, which is upregulated by PPAR*γ*, can combine with Bax and Beclin-1 to reduce apoptosis and autophagy, respectively. Thus, fucosterol attenuates Concanavalin A-induced acute liver injury in mice via the P38 MAPK/PPAR*γ*/NF-*κ*B pathway.

PPAR*γ* is a member of the nuclear transcription factor superfamily that is activated by corresponding ligands and combines with retinoid X receptor (RXR) to form a distinct dimer that regulates transcription [13]. Van Ginderachter et al. and Ricote et al. showed that PPAR*γ* is a negative regulator of macrophage activation [12, 33]. In inflammatory responses, it can bind NF-*κ*B p65/p50 to form an inhibitory transcriptional complex that reduces the expression of related inflammatory factors [6, 11, 34]. Additionally, Ren et al. and Fuenzalida et al. provided evidence that PPAR*γ* upregulates Bcl-2, preventing oxidative stress-induced neuronal and cardiomyocyte degeneration [8, 31]. Our results showed that PPAR*γ* expression and NF-*κ*B p65 activation were increased to different degrees after treatment, indicating that fucosterol might be a potential PPAR*γ* activator. However, there is no evidence that fucosterol binds directly to PPAR*γ*. Based on existing studies, it is believed that MAPK-mediated PPAR*γ* phosphorylation contributes to reduced PPAR*γ* transcriptional activity and, thereby, inhibits oxidative injury and inflammation [32, 35]. Fucosterol is associated with the MAPK family [16, 36], therefore, we examined the levels of P38 MAPK, ERK, and JNK phosphorylation. These results showed that although ConA increased the phosphorylation of all three components, fucosterol acted only on the phosphorylation of P38 MAPK in this model (Figure 6). Interestingly, Lee et al. revealed that fucosterol inhibited adipogenesis of 3T3-L1 preadipocytes by modulating FoxO signaling, in which PPAR*γ* was inactivated [37]. These differences could be attributed to the different pathogenesis of the various disease models.

## 5. Conclusions

Our findings showed that fucosterol alleviated ConA-induced acute liver injury via the P38 MAPK/PPAR*γ*/NF-*κ*B pathway. Our data show that fucosterol attenuated serum liver enzyme levels by inhibiting necrosis and apoptosis in a process mediated by PPAR*γ* activation and NF-*κ*B inhibition, which reduced the release of inflammatory factors. Fucosterol also inhibited apoptosis and autophagy by upregulating Bcl-2 via PPAR*γ*, which reduced the levels of functional Bax and Beclin-1. These findings highlight fucosterol as a promising potential therapeutic agent for AIH.

## Figures and Tables

**Table 1:** Nucleotide sequences of primers used for qRT-PCR.

Gene		Primer sequence (5′-3′)
TNF-*α*	Forward	CAGGCGGTGCCTATGTCTC
	Reverse	CGATCACCCCGAAGTTCAGTAG
IL-1*β*	Forward	GCCACGGCACAGTCATTGA
	Reverse	TGCTGATGGCCTGATTGTCTT
IL-6	Forward	CTGCAAGAGACTTCCATCCAG
	Reverse	AGTGGTATAGACAGGTCTGTTGG
Beclin-1	Forward	ATGGAGGGGTCTAAGGCGTC
	Reverse	TGGGCTGTGGTAAGTAATGGA
P62	Forward	GAGGCACCCCGAAACATGG
	Reverse	ACTTATAGCGAGTTCCCACCA
LC3-II	Forward	GACCGCTGTAAGGAGGTGC
	Reverse	AGAAGCCGAAGGTTTCTTGGG
PPAR*γ*	Forward	GGAAGACCACTGCATTCCTT
	Reverse	GTAATCAGCAACCATTGGGTCA
Bax	Forward	AGACAGGGGCCTTTTTGCTAC
	Reverse	AATTCGCCGGAGACACTCG
Bcl-2	Forward	GCTACCGTCGTCGTGACTTCGC
	Reverse	CCCCACCGAACTCAAAGAAGG
*β*-Actin	Forward	GGCTGTATTCCCCTCCATCG
	Reverse	CCAGTTGGTAACAATGCCATGT
